# Recovery of Chronic Stress-Triggered Changes of Hippocampal Glutamatergic Transmission

**DOI:** 10.1155/2018/9360203

**Published:** 2018-01-30

**Authors:** Min Lin, Gonglin Hou, Ying Zhao, Ti-Fei Yuan

**Affiliations:** ^1^Department of Psychology, Zhejiang Sci-Tech University, Hangzhou, Zhejiang, China; ^2^Shanghai Key Laboratory of Psychotic Disorders, Shanghai Mental Health Center, Shanghai Jiao Tong University School of Medicine, Shanghai, China; ^3^Co-innovation Center of Neuroregeneration, Nantong University, Nantong, Jiangsu, China

## Abstract

Chronic stress results in neurochemical, physiological, immune, molecular, cellular, and structural changes in the brain and often dampens the cognition. The hippocampus has been one major focus in studying the stress responsivity and neural mechanisms underlying depression. Both acute and chronic stress stimuli lead to dynamic changes in excitatory transmission in the hippocampus. The present study examined the potential effects of spontaneous recovery after chronic stress on spatial memory function and glutamatergic transmission in the hippocampus. The results showed that chronic unpredicted mild stress transiently increased AMPA receptor GluA2/3 subunit expression, together with elevated PICK-1 protein expression. Spontaneous recovery restored the behavioral deficits in Barnes maze test, as well as the glutamate receptor expression changes. In conclusion, spontaneous recovery acts as an important mechanism in system homeostasis.

## 1. Introduction

Chronic stress results in neurochemical, physiological, immune, molecular, cellular, and structural changes in the brain and often dampens the cognition [1–4]. The hippocampus is believed to be responsible for the decreased learning and memory abilities following chronic stress. It has been found that chronic stress decreases adult hippocampal neurogenesis, blocks LTP induction, downregulates expression of neurotrophic factors, and exacerbates neuronal apoptosis in the hippocampus [5–7]. In addition, chronic stress alters glia homeostasis [8–10], such as triggering microglial cell proliferation and activation, suppresses astrocyte proliferation, and decreases the expression of GFAP protein and excitatory amino acid transporters (EAATs) in astrocytes, which might contribute to the altered excitatory transmission (e.g., glutamatergic) of hippocampal neurons following chronic stress. Limited evidences revealed that suppressed hippocampal neurogenesis is recovered following removal of stress [11].

The hippocampus has been one major focus in studying the stress responsivity and neural mechanisms underlying depression. The excitatory transmission in the hippocampus is mainly mediated by glutamatergic synapses, with two types of inotropic glutamate receptors: AMPA receptor and NMDA receptor, respectively. AMPA receptors are composed of GluA1–4 subunits, which bind to scaffolding proteins postsynaptically to be functional on the membrane. PICK-1 and PSD-95 interact with the glutamatergic receptors, regulating their membrane distribution and functions [12–14].

Acute stress potentiates the AMPA receptor transmission in the hippocampus, inducing insertion of GluA2-lacking AMPA receptors in the CA1 region (reflected by increased AMPA/NMDA ratio and lack of change in NMDA-mEPSCs) [15, 16], while chronic stress might impair or have no effect on AMPA transmission in the hippocampus [17, 18]. In the present study, we examined the potential effects of spontaneous recovery after chronic stress on spatial memory function and glutamatergic transmission in the hippocampus. The results suggested that spontaneous recovery might act as an important endogenous mechanism in self-repair.

## 2. Materials and Methods

### 2.1. Ethics

The study has been approved by Ethics Committee of Animal Experiments in Zhejiang Sci-Tech University, and all procedures followed the guidelines to animal experiments in Zhejiang Sci-Tech University.

### 2.2. Animals

36 male Sprague-Dawley rats were purchased from Animal Experiment Center, Chinese academy of Sciences, and raised under temperature 22 ± 2°C, humidity 50–60%, 12/12 hours L/D cycle, and free access to food/water. All animals were subjected for a 10-day adaptation period before being randomly assigned into control group, model (chronic stress) group, or recovery (recovery from chronic stress) group (12 in each group). The control group animals were housed as 3 rats per cage, while the CUMS rats were housed alone and subjected to chronic unpredictable mild stress (CUMS) of 35 days. For the recovery group, the animals received CUMS and then left for recovery of another 35 days.

### 2.3. Procedures for CUMS

CUMS procedures included as follows: food deprivation (24 hours), water deprivation (24 hours), tail pinch (1 minute), food shock (1.0 mA for 10 seconds, 30 times with interval of 1 minute), ice water swimming (4°C, 5 minutes), wet bedding (24 hours), and reversed light/dark cycle. The seven types of stress were randomly presented to animals with each per day [6].

### 2.4. Open Field Test

Open filed is 80 cm × 80 cm × 40 cm (length, width, and height), and the central field was set as 40 cm × 40 cm in Noldus software (Noldus Co., Netherlands). The animal was let freely in the open field for 5 minutes.

### 2.5. Barnes Maze Test

Barnes maze was built as previously described [19]. The maze is composed of a 122 cm diameter plate with 18 holes (diameter 10 cm) under bright light. One dark box was under the plate, and the animal can hide into the box through one of the hole on the plate. For each test, the plate was turned while the dark box was in fixed position. The animal was trained once per day for three continuous days. In the test phase, the animal was placed in the central of the plate with random head direction and the behavior trace was recorded.

### 2.6. Corticosterone Measurement

1 ml cardiac blood was sampled in the morning and centrifuged at 3000 r/min under 4 degrees Celsius for serum isolation. The supernatant was kept at −80 degrees before measuring with Corticosterone ELISA kit (Abcam). The procedure was performed following the kit brochure, and the corticosterone concentration was calculated from the standard curve.

### 2.7. Western Blot

The acutely dissociated hippocampus tissue was weighted and homogenized for protein extraction. The primary antibody was mice-anti-Rat GluA1/2/3/PICK-1/PSD-95 (Millipore, 1 : 100). The blot was finally revealed with ECL system and imaged in Tanon-2500 system.

### 2.8. Immunohistochemistry

The animals were sacrificed, and brains were harvested for paraffin embedding, sectioning, and immunostaining (or HE staining). Briefly, the sections were firstly incubated with primary antibody mice-anti-Rat GluA1/2/3/PICK-1/PSD-95 (Millipore, 1 : 400) overnight at 4 degrees, then secondary antibody goat-anti-mice IgG conjugated for Envision kit (1 : 400, Boshide, Wuhan, China). The images were taken under a Zeiss fluorescence microscope and analyzed by Image-Pro plus software (Media Cybernetics, USA). For stereological analyses, six sections of the hippocampus were chosen from each side (Bregma −3.3, −3.6, −3.9, −4.2, −4.5, and −4.8 mm). The boundary of the hippocampus was defined as previously described [20].

### 2.9. Statistics

The data were presented as mean ± SD and analyzed with SPSS 13.0 software (Chicago, USA). The differences between two groups were compared by independent sample *t*-test and ANOVA for the three groups. *P* < 0.05 was considered as statistically significant.

## 3. Results

### 3.1. Serum Corticosterone Response and Behavior Changes

Chronic stress induced significant elevation in serum corticosterone concentration of the CUMS group (101.4 ± 20.3 ng/ml), compared to the control group (67.7 ± 15.4 ng/ml) (*F*_(2,19)_ = 12.233, *P* < 0.01). In the recovery group, the corticosterone concentration (59.6 ± 11.2 ng/ml) went back to control level.

**Figure 1:**
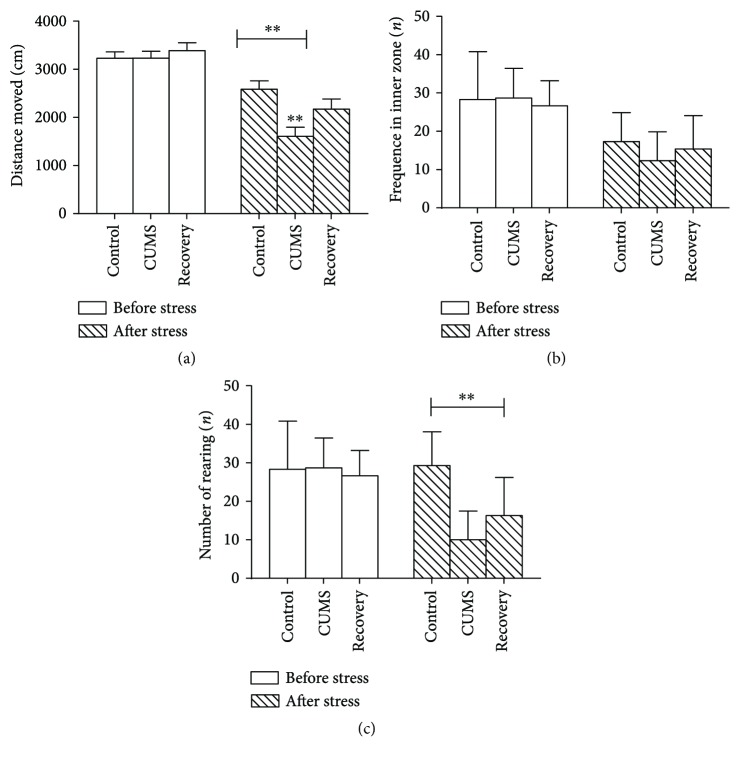
Open field test results. (a) The animals in the CUMS group exhibited decreased movement after stress, which was restored in the recovery group. (b) There was no significant difference among the three groups in frequencies crossing the inner zone. (c) The animals in the CUMS group exhibited decreased vertical rearing exploration after stress, which was restored in the recovery group. ^∗∗^*P* < 0.01.

In the open field test, the CUMS group animals exhibited decreased locomotion in the maze, as well as decreased vertical rearing (Figures 1(a) and 1(c)), but not the frequencies of central crosses (Figure 1(b)). In addition, the recovery group animals rehabilitated and demonstrated restored behavior responses (Table 1).

**Figure 2:**
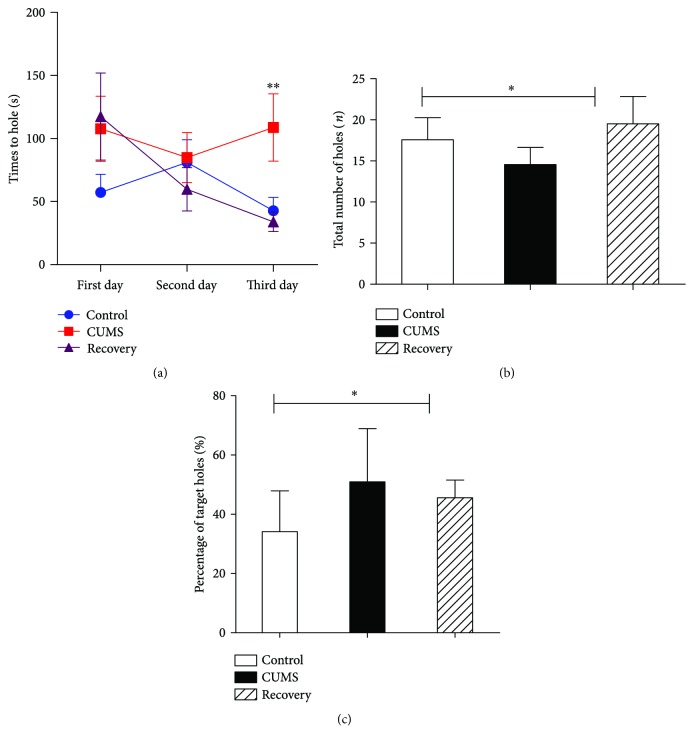
Barnes maze test results. (a) The animals in the CUMS group exhibited decreased learning ability in Barnes maze test, which was restored in the recovery group. (b) In the test phase, the animals in the CUMS group exhibited decreased exploration ability (reflected by total number of holes explored). (c) In the test phase, the CUMS group animals exhibited deficits in short-term working memory (reflected by repeated exploration of the target hole). ∗ suggests for *P* < 0.05 and ∗∗ for *P* < 0.01.

In the Barnes maze test, the CUMS group showed decreased ability of learning in the first 3 days (Figure 2(a)):one-way ANOVA revealed significant effect (*F*_(2,27)_ = 7.277, *P* < 0.01). In the test phase, the CUMS group explored fewer holes than the control group and spent more time in the target hole area (Figure 2(b), one-way ANOVA (*F*_(2,27)_ = 8.125, *P* < 0.01); Figure 2(c), one-way ANOVA (*F*_(2,27)_ = 3.490, *P* < 0.05)), suggesting for impaired short-term memory and spatial learning ability. On the other hand, the recovery group exhibited control level performance in the test.

### 3.2. Expression Changes of AMPA Receptor Subunits

**Figure 3:**
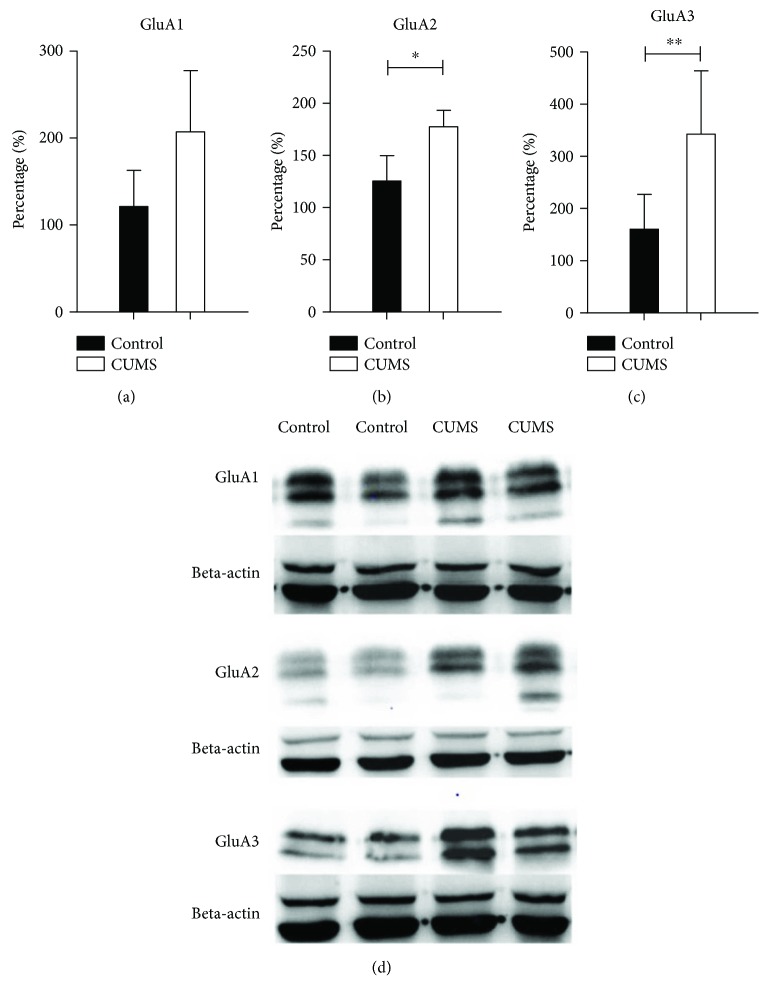
Western blot results of GluA1–3 subunit expression in the whole hippocampus. There was no clear increase of GluA1 subunit, while the expression levels of GluA2/3 subunits were significantly upregulated. ∗ suggests for *P* < 0.05 and ∗∗ for *P* < 0.01.

AMPA receptors are important components in excitatory transmission. We therefore examined the changes of AMPA receptors subunits GluA1–3 in the hippocampus following chronic stress. With Western blot of full hippocampus tissue, we found that in the CUMS group, the expression levels of GluA2 (independent sample *t*-test, *t* = 6.893, *P* < 0.05, *n* = 8) and GluA3 (independent sample *t*-test, *t* = 12.966, *P* < 0.01, *n* = 8) subunits were significantly elevated, but not the GluA1 subunit (independent sample *t*-test, *t* = 4.381, *P* = 0.08, *n* = 8) (Figure 3).

**Figure 4:**
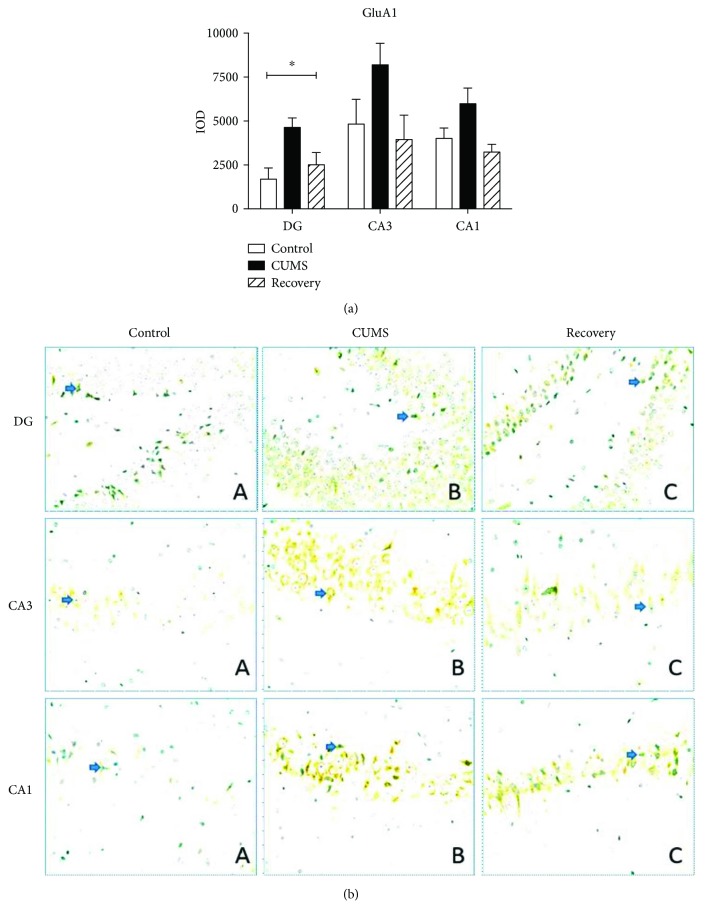
Immunohistochemistry results showing the expression of GluA1 in the subregion of the hippocampus. ^∗^*P* < 0.05.

We further compared the expression changes of GluA1–3 subunits in the subregion of the hippocampus (DG, CA3, and CA1) with immunohistochemistry and semiquantitative measurement by optic density. For GluA1 subunit, we found that the expression in the DG region is upregulated in the CUMS group when compared to the control (one-way ANOVA, *F*_(2,25)_ = 3.491, post hoc test, *P* < 0.05), but not in the CA3 (*F*_(2,25)_ = 2.991, *P* = 0.063) and CA1 regions (*F*_(2,25)_ = 1.861, *P* = 0.17). In addition, the recovery group showed restored level of GluA1 expression (Figure 4).

**Figure 5:**
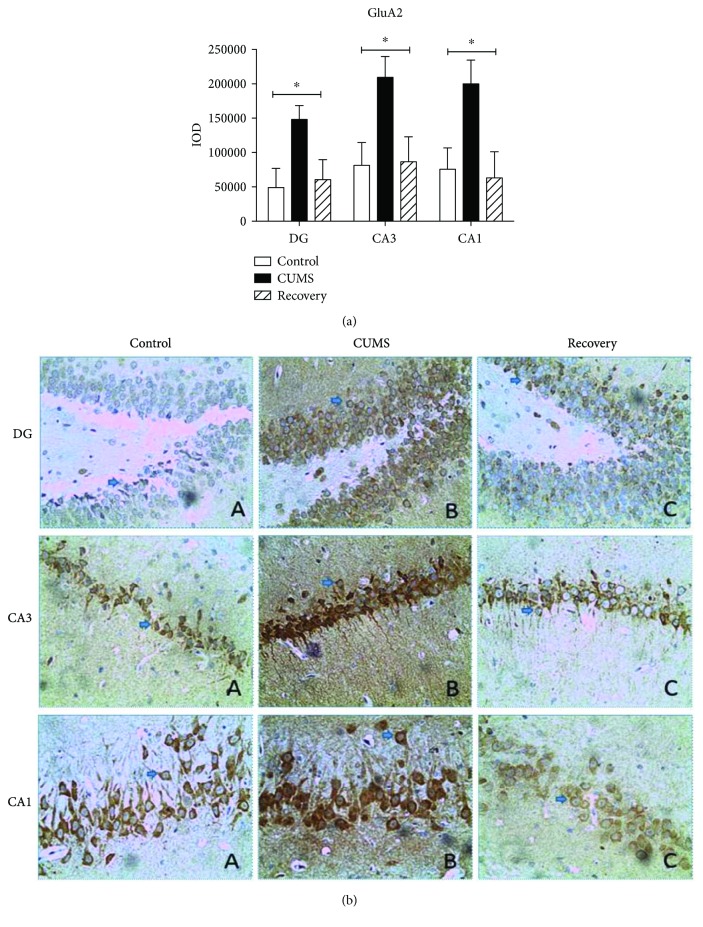
Immunohistochemistry results showing the expression of GluA2 in the subregion of the hippocampus. ^∗^*P* < 0.05.

**Figure 6:**
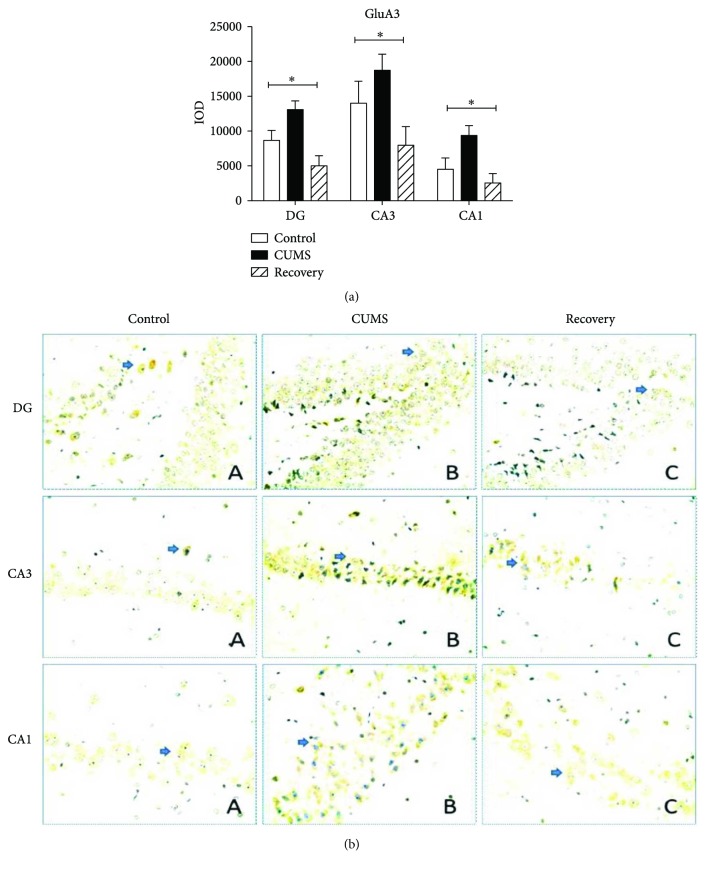
Immunohistochemistry results showing the expression of GluA3 in the subregion of the hippocampus. ^∗^*P* < 0.05.

For GluA2 subunits, in the DG, CA3, and CA1 regions, we all found the upregulated expression in the CUMS group, compared to the control group (post hoc test, *P* < 0.05) and the recovery group (post hoc test, *P* < 0.05) (Figure 5). Compared by one-way ANOVA, CUMS increased GluA2 expression in DG (*F*_(2,31)_ = 3.729, *P* < 0.05), CA3 (*F*_(2,31)_ = 4.658, *P* < 0.05), and CA1 (*F*_(2,31)_ = 4.406, *P* < 0.05). Meanwhile for GluA3 subunits, compared to the control group, one-way ANOVA reveals a significant effect of CUMS in the DG/CA1 regions (in the DG region, *F*_(2,23)_ = 9.102, *P* < 0.01, post hoc test, *P* < 0.01; in the CA1 region, *F*_(2,23)_ = 5.364, *P* < 0.01, post hoc test, *P* < 0.01) and in the CA3 region (*F*_(2,23)_ = 4.629, *P* < 0.05, post hoc test, *P* < 0.05). In addition, compared with the CUMS group, the recovery group exhibited restored level of GluA3 expression in the DG and CA1 regions (post hoc test, *P* < 0.01) (Figure 6).

### 3.3. Expression Changes of AMPA Receptor-Associated Proteins

**Figure 7:**
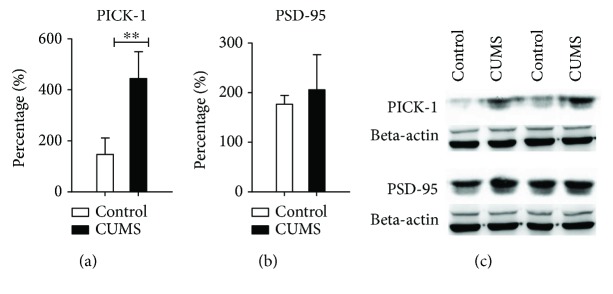
Western blot results of PICK-1 and PSD-95 expression in the whole hippocampus. There was no clear change for PSD-95 expression, while the expression level of PICK-1 was significantly upregulated. ^∗∗^*P* < 0.01.

In order to explore the potential molecular mechanism underlying AMPA receptor subunit changes, we then examined the postsynaptic scaffolding proteins that bind to AMPA receptor, such as PICK-1 and PSD-95. With Western blot of full hippocampus tissue, we found that in the CUMS group, the expression level of PICK-1 (independent sample *t*-test, *t* = 23.260, *P* < 0.01, *n* = 8) protein was significantly elevated compared to the control group, but not the PSD-95 protein (independent sample *t*-test, *P* = 0.34) (Figure 7).

**Figure 8:**
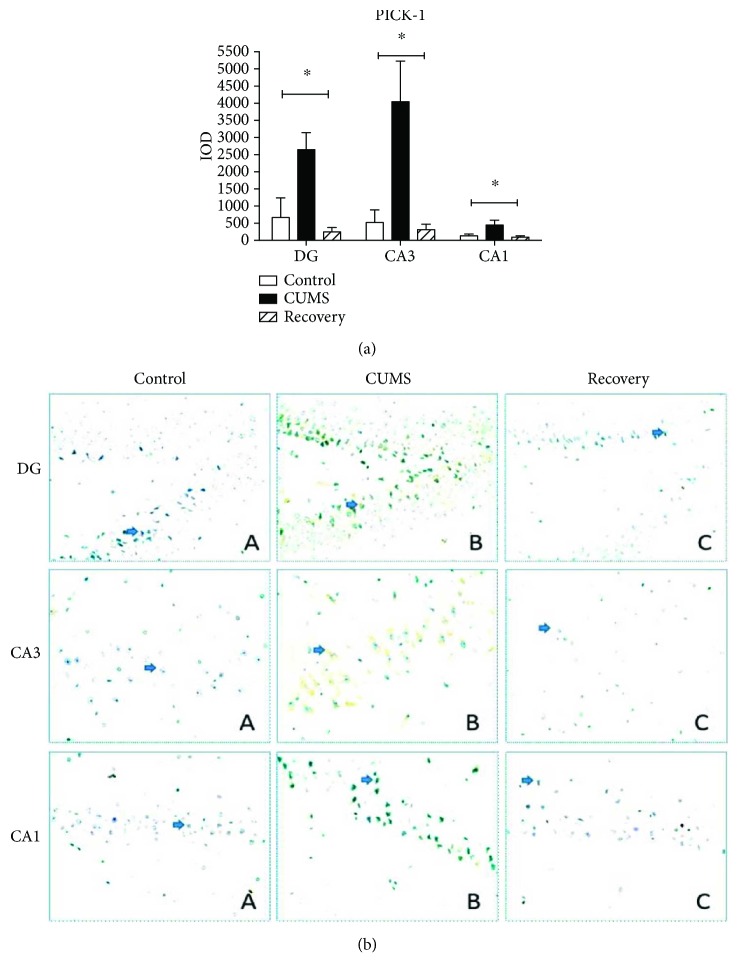
Immunohistochemistry results showing the expression of PICK-1 in the subregion of the hippocampus. ^∗^*P* < 0.05.

**Figure 9:**
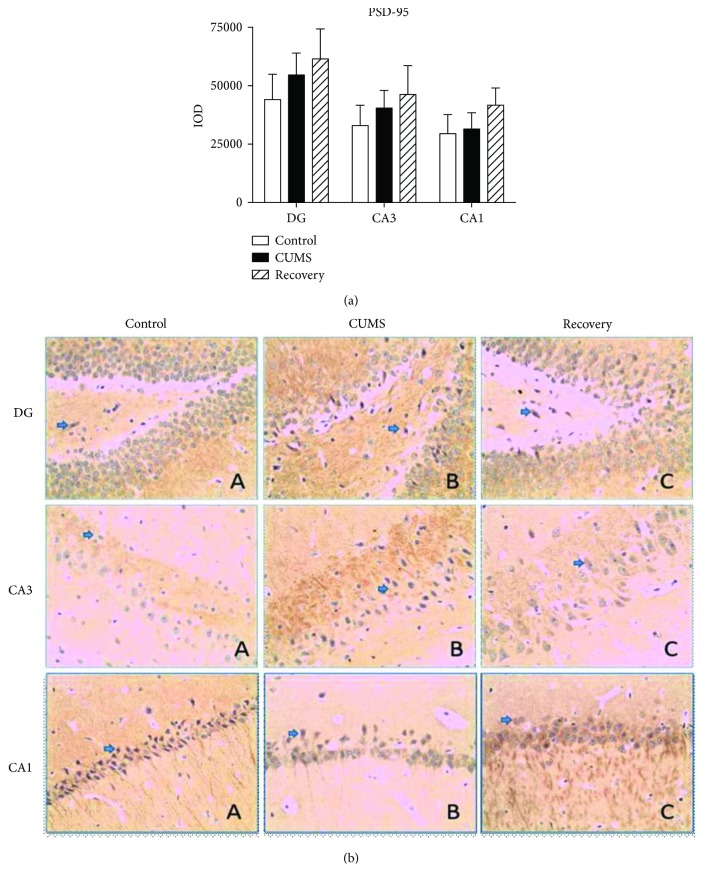
Immunohistochemistry results showing the expression of PSD-95 in the subregion of the hippocampus.

We further compared the expression changes of PICK-1 and PSD-95 in the subregion of the hippocampus (DG, CA3, and CA1) with immunohistochemistry and semiquantitative measurement by optic density. For PICK-1, one-way ANOVA analysis in the DG and CA1, respectively, yielded *F*_(2,37)_ = 6.013 (*P* < 0.01) and *F*_(2,37)_ = 17.219 (*P* < 0.01), while in the CA3 region, it yielded *F*_(2,37)_ = 4.805 (*P* < 0.05) all exhibiting a significant different expression among the three groups. There was an upregulated expression of PICK-1 in the CUMS group compared to the control group (post hoc test, in DG/CA3, *P* < 0.05, and in CA1, *P* < 0.01) and the recovery group (post hoc test, in the DG/CA1, *P* < 0.01, and in CA3, *P* < 0.05) (Figure 8). On the other hand, in line with the Western blot result, there was no significant change of PSD-95 expression across all groups (Figure 9).

## 4. Discussion

Disrupted glutamatergic transmission is reported in both animal models of depression and postmortem brain from depression patients [21]. We here showed that, together with the restored brain function after a recovery period after chronic stress, the glutamatergic transmission is also recovered. The hippocampus has been one major focus in studying the stress responsivity and neural mechanisms underlying depression. Acute stress potentiates the AMPA receptor transmission in the hippocampus, inducing insertion of GluA2-lacking AMPA receptors in the CA1 region (reflected by increased AMPA/NMDA ratio and lack of change in NMDA-mEPSCs) [15, 16], while chronic stress might impair or have no effect on AMPA transmission in the hippocampus [17, 18, 22]. In present study, we reported that the expression of AMPAR subunits were increased following chronic stress, which decreased to baseline following a period of recovery, in line with the behavioral restoration.

In our results, we detected significant elevation of GluA2/3 expression in the hippocampus, but not GluA1. This suggested for insertion or synthesis of GluA2-containing AMPA receptors, which might act in replacement of GluA2-lacking AMPA receptors that are recruited in acute stress phases. However, one limitation of the study is that we did not discriminate synaptic and somatic AMPA receptors and if these receptors are involved in synaptic transmission. It should also be noted that the receptor changes could be pathway-specific in CA1 [17], which requires electrophysiological investigations (such as measuring the synaptic strength in projection-specific manner) in the future.

PICK-1 is important in AMPA receptor trafficking during long-term synaptic plasticity [12, 13]. In fact, PICK-1 interacts with GluA2 subunit directly and is involved in the incorporation of GluA2-containing AMPA receptors as the replacement of GluA2-lacking AMPA receptors [23]. The expression level of PICK-1 was tightly linked to the expression of GluA2 subunit [24], as well as the strength of AMPA receptor transmission [14]. The present study reported the elevation of PICK-1 protein in the CUMS group, which is consistent with the previous data on AMPA receptor subunits. In addition, the results indicate that the upregulated AMPA receptors are functional, since they interact with PICK-1 at postsynaptic site. On the other hand, we did not detect clear changes in PSD-95 proteins. It is possible that they are in much higher quantity compared to AMPA receptor, and therefore the AMPA receptor trafficking did not affect their expression significantly.

In conclusion, recovery of chronic stress is able to restore the glutamatergic transmission in the hippocampus, as well as the impaired cognitive behaviors. In the future study, it is yet to confirm investigate the functional aspects of AMPA receptor expression changes, such as with electrophysiological approaches.

## Figures and Tables

**Table 1:** The results of open field test (M ± SD).

	Locomotion (cm)	Central cross times	Rearing times
Control (*n* = 12)	2585.1 ± 173.0	17 ± 8	29.3 ± 8.8
CUMS (*n* = 10)	1605.5 ± 189.5^∗∗^	12 ± 8	10.0 ± 7.4^∗∗^
Recovery (*n* = 8)	2171.2 ± 211.8	15 ± 9	16.3 ± 9.9
*F*	7.297	1.115	14.065
*P* value	0.003	0.343	0.000

^∗∗^ *P* < 0.01.
